# Bonding of Flexible Membranes for Perfusable Vascularized Networks Patch

**DOI:** 10.1007/s13770-021-00409-1

**Published:** 2021-12-06

**Authors:** Soyoung Hong, Yejin Song, Jaesoon Choi, Changmo Hwang

**Affiliations:** 1 Biomedical Engineering Research Center, Asan Institute for Life Sciences, Asan Medical Center, 88 Olympic-ro 43-gil, Songpa-gu, Seoul, 05505 Republic of Korea; 2 Department of Biomedical Engineering, Asan Medical Center, University of Ulsan College of Medicine, 88 Olympic-ro 43-gil, Songpa-gu, Seoul, 05505 Republic of Korea

**Keywords:** Membrane bonding, Vascular networks, Complex tissue generation, Perfusion culture

## Abstract

**BACKGROUND:**

*In vitro* generation of three-dimensional vessel network is crucial to investigate and possibly improve vascularization after implantation *in vivo*. This work has the purpose of engineering complex tissue regeneration of a vascular network including multiple cell-type, an extracellular matrix, and perfusability for clinical application.

**METHODS:**

The two electrospun membranes bonded with the vascular network shape are cultured with endothelial cells and medium flow through the engineered vascular network. The flexible membranes are bonded by amine-epoxy reaction and examined the perfusability with fluorescent beads. Also, the perfusion culture for 7 days of the endothelial cells is compared with static culture on the engineered vascular network membrane.

**RESULTS:**

The engineered membranes are showed perfusability through the vascular network, and the perfused network resulted in more cell proliferation and variation of the shear stress-related genes expression compared to the static culture. Also, for the generation of the complex vascularized network, pericytes are co-cultured with the engineered vascular network, which results in the Collagen I is expressed on the outer surface of the engineered structure.

**CONCLUSION:**

This study is showing the perfusable *in vitro* engineered vascular network with electrospun membrane. In further, the 3D vascularized network module can be expected as a platform for drug screening and regenerative medicine.

**Supplementary Information:**

The online version contains supplementary material available at 10.1007/s13770-021-00409-1.

## Introduction

A vascular bed is an intricate network of nutritive blood vessels that ramifies through the tissues of the body or of one of its parts. The vascular network in the bed is vital for the efficient transport of oxygen and nutrients to tissue and metabolic waste exchanges take place by diffusion [1]. An important goal in the field of tissue engineering is the generation of three-dimensional (3D) vascularized tissue. Several recent studies have reported potential vascularization techniques in 3D engineered constructs.

Natural tissue vascularization is a complex process and takes time that develops through vasculogenesis and angiogenesis *in vivo* [2, 3]. The natural blood vessels formed by vasculogenesis or angiogenesis are eventually remodeled and matured as per the demands of specific tissues through the upregulation of various growth factors. The strategies of a large tissue regeneration rely on host-capillary invasion mediated by angiogenesis and vasculogenesis on tissue constructs post-implantation. The engineered tissues grown in the laboratory have limitations in the diffusion of host cells following a slow rate of host-capillary migration when implanted into the body [4]. Thus, a pre-vascularized tissue is considered critical before implantation for integration with host vasculature *in vivo*.

In order to generate a 3D pre-vascularized structure, the researchers have tried a simple method of making vascular endothelial cells by mixing with hydrogel [5, 6] and a method with complex and controllable methods such as 3D printing [7–10] and micro-patterning chip [11]. Also, decellularization of the natural tissue was showed to mimic the native vascular network *in vitro* [12]. In the use of 3D printing, a sacrificial mold was printed to have the shape of a grid [13, 14] or branched vascular tree [15, 16], and then the sacrificial mold was encapsulated with hydrogel. Jordan et al. showed that after printing using carbohydrate glass in a lattice form, a perfusable engineered 3D tissue was produced using polymeric hydrogel [14]. In the case of Zhang et al., a polymeric hydrogel was used to construct a perfused vascular network structure that can be applied *in vivo* [15]. Also, in the microfluidic chip, a collagen-based microfluidic chip was produced to create a vascular network *in vitro*, and drug toxicity tests were performed [13, 17, 18].

To generate a 3D vascular network *in vitro* for application *in vivo*, hydrogel or synthetic scaffolds are used to provide regenerative cues that induce formation and maintenance of the vascular bed, as well as encase and retain the network of engineered microvessels *in vitro*. Vascular network scaffold using hydrogel has advantages with their biological compatibility, tunable biodegradability, and porous structure. However, hydrogel-based constructs are limit their utilization in many applications that require high stress especially for *in vivo* applications, because of mechanical properties, swelling and degradation in hydrogels in a short time, and low mechanical strength and fragile nature [19]. Also, it is difficult to tune the mechanical properties of the only natural hydrogel to match the hardness of the vascular channel and maintain the culture *in vitro* with the perfusion of a vascular channel within the hydrogel.

The synthetic polymer material has been used as a blood vessel substitute for decades *in vivo*. Among the synthetic polymers, the polymeric electrospun membrane has properties that controllable nanofiber [20] for cell attachment, porosity, mechanical property, and controllable degradation for a long-term culture or implantation, and the nanofibers are similar with extracellular matrix (ECM) interwoven fiber network [20]. Also, the electrospun membrane has the porosity for permeability and communication with outer fibroblasts or growth factors. However, the flexible electrospun membrane is a stumbling block for the generation of thick 3D and branchy vessel structures. The flexible membrane with a vascular network is available for skin necrosis and ischemic lesions such as diabetic foot ulcers having the capability of delivery of oxygen and nutrient. And then the vascular network membrane could be combined with other natural or synthetic scaffolds for complex tissue generation.

Our research is already reported that the stacking of embossed membranes could be a guide for fabrication vasculogenesis in 3D stacked membranes [21]. However, *in vitro* culture with an embossed membrane-embedded, vascular network was not conducted owing that the vascular channel was not fabricated *in vitro*, and vasculogenesis was conducted after implantation of the embossing membranes. Also, pre-vascular networks forming with electrospun membrane and perfusion culture *in vitro* have not been researched as far as we know.

In this work, we aim at developing a 3D vascular network within the electrospun membrane utilized able to recapitulate for bonding of two-dimensional polymer membrane for application *in vivo*. The generated vascular network was confirmed perfusability, and then endothelial cells were adhered and cultured onto the inner surface of the generated vascular network. The perfusion cultured vascular cells of the vascular network were confirmed adhesion for EC monolayer and proliferation with immunofluorescence staining and analyzed by rt-PCR. We hypothesize that the perfusion of the culture medium through the vessels of membranes will foster the growth of the EC.

## Materials and methods

### Vascular pattern molding with alginate

A master mold with vascular network patterns of SU-8 1000 µm height was prepared by MicroFIT (Seongnam, South Korea) (Supplementary Fig. S1, Polydimethylsiloxane (PDMS) (Dow Corning, Seoul, South Korea) pre-polymer mixture was prepared at a 10:1 base to curing agent. The PDMS solution was cast onto the SU-8 mold and thermally cured at 60 °C for 2 h to obtain a negative replica-mold with vascular network channels 1000 µm in height. Gelatin (10% w/v, Sigma, Seoul, South Korea) in CaCl_2_ solution (5% w/v, Sigma) was casted in the Petri dish (SPL, Pocheon, South Korea), and then the gelatin solution was gelled in the 4 °C for 1 h. The negative PDMS mold was placed onto the gelatin hydrogel, and an alginate solution (6% w/v) was injected into the PDMS mold and crosslinked for one hour. The casted alginate vascular network mold was incubated in CaCl_2_ (5% w/v) solutions for 1 h and then separated from the PDMS mold. The fabricated alginate mold was inserted into a silicone tubing (Quosina, Ronkonkoma, NY, USA) at both sides for connections with a pump (Supplementary Fig. S2).

### Bonding of electrospun membranes with vascular pattern

**Fig. 1:**
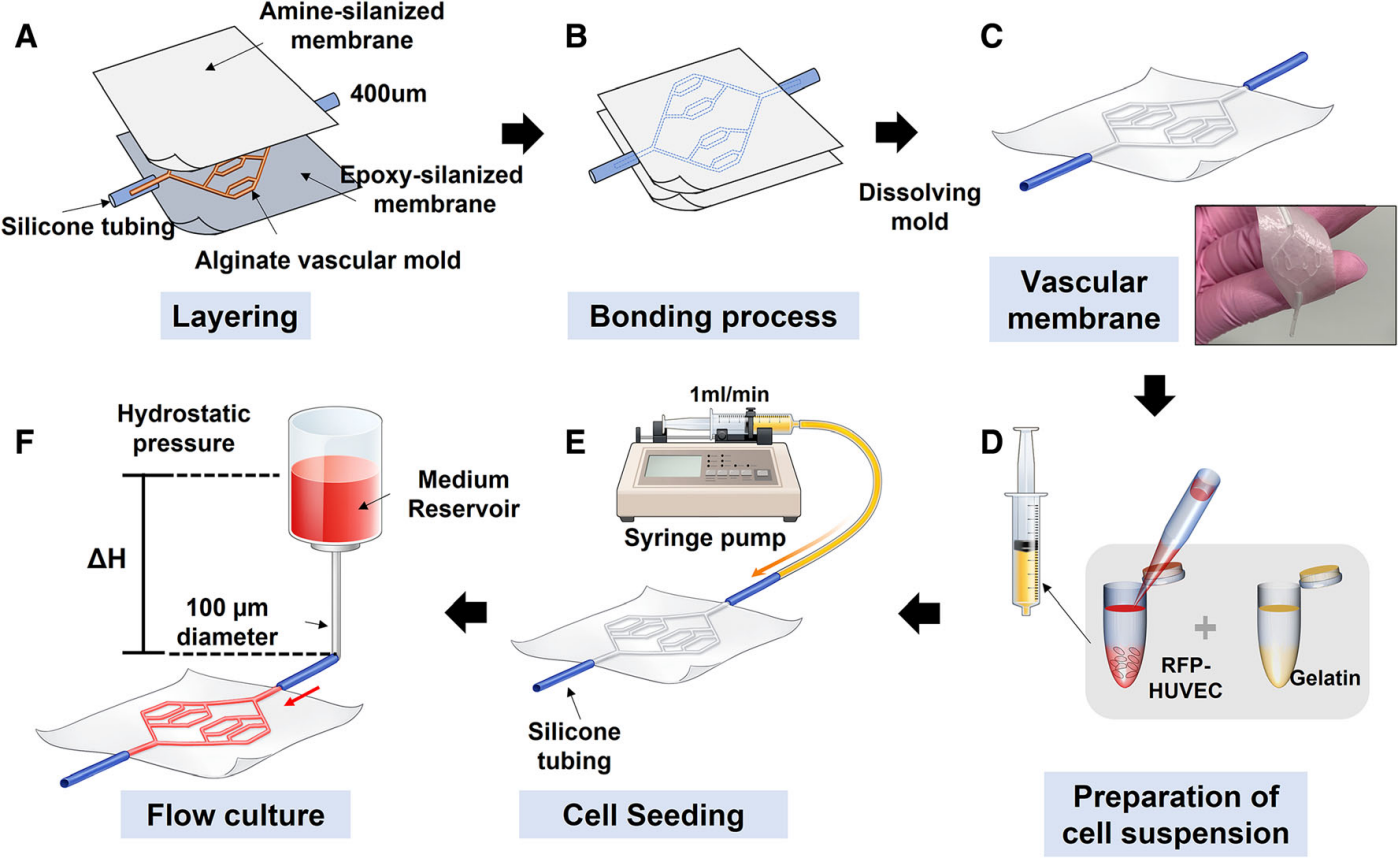
Fabrication of vascular network channel within the electrospun membrane **A** layering of two membranes and alginate vascular network mold. **B** Embossing forming and bonding with heat and pressure and **C** bonded embossed electrospun membrane within vascular network sacrificial mold and dissolving alginate vascular network mold. **D** Preparation of R-HMVEC solution in 3% (w/v) gelatin solution for cell seeding. **E** Injection of cell suspension using a syringe pump. **F** Perfusion culture with hydrostatic pressure for a vascular network channel

An embossed electrospun membrane was created using a vacuum forming method on Poly (l-lactic acid-co-ε-caprolactone) (PLCL, Evonik, Essen, Germany) electrospun membranes. PLCL membrane was prepared as a previous study [22]. A solution of PLCL (12.5% w/v) in a solvent mixture (40:60 of dimethylformamide (DMF, Daejung, Siheung, South Korea) and tetrahydrofuran (THF, Daejung) was dissolved with stirring at 80 °C for at least 4 h. The PLCL solution feed was driven by a syringe pump at a flow rate of 2 mL/h, and an 18-cm working distance and direct current (DC) voltage of 20 kV was applied between the needle tip and aluminum foil collector using an electrospinning machine (NanoNC Co., Ltd, Seoul, South Korea). The electrospun polymer membrane was collected in the form of a mesh on the collector with 2000 rpm. The collected PLCL membrane was dried overnight in a fume hood. Figure 1 shows the schematic process of chemical bonding and embossing process at the same time. First, the surfaces of the PLCL membranes were treated with 20 sccm oxygen (O_2_) plasma for 30 s. 2% (3-Aminopropyl) triethoxysilane (APTES, Sigma) or 2% (3-glycidyloxypropyl) trimethoxysilane (GPTMS, Sigma) in methanol (99.9%) were poured onto the plasma-treated PLCL membranes for 2 h. After silanization, APTES- or GPTMS- anchored membranes were washed with methanol and dried completely with air-drying. The pre-made alginate vascular network mold was lied between two surface-treated PLCL membranes. After layering of the alginate mold and two surface-treated PLCL membranes, bonding and vacuum formation of PLCL membranes were carried out using a homemade system, as described in a previous study [21]. The membrane-alginate-membrane set was laid directly into the homemade chamber, and a vacuum (-0.56 bar) was applied at 100 °C for 4 h to assist bonding. After the bonding process, only the two membranes were bonded; the alginate sacrificial mold was removed by dissolving in trisodium citrate dihydrate (0.2 M, Sigma) for 24 h at RT.

### Surface characterization

#### XPS analysis

The bonded electrospun membranes were observed by scanning electron microscopy (AIS2000C, Seron Technologies, Uiwang, South Korea). The silanized surface of the membrane was characterized by X-ray photoelectron spectroscopy (XPS) analysis using an AXIS SUPRA (Kratos, Manchester, UK). The pressure in the chamber was below 5 × 10^–10^ Torr before the data was taken, the voltage was 20 kV. XPS analyses were interpreted using ESCAII.

#### Fluorescence measurement

The silanized surface was further confirmed by fluorescence measurement using amine- and carboxylate-modified fluorospheres. Amine-modified fluorospheres (0.2 µm, Thermo Fisher Scientific, Seoul, South Korea) were diluted 50-fold with distilled water, and carboxylate-modified fluorospheres (0.2 µm, Thermo Fisher Scientific) were diluted 50-fold in a 1:1 (v/v) mixture solution of N′-ethyl carbodiimide hydrochloride (150 mm, EDC, Sigma) and N-hydroxysuccinimide (60 mm, NHS, Sigma), both prepared in Phosphate-Buffered Saline (PBS, pH 7.4, Thermo Fisher Scientific), to enhance the binding of the carboxylate-functionalized fluorospheres with amine functionality by activating the carboxyl acid (–COOH) group. After 30 min, the excess EDC and NHS were removed by rinsing with PBS buffer and then with distilled water. Fluorescence was observed using a JULI stage (NanoEntek, Seoul, South Korea).

### Bonding strength analysis

The bonding strength of bonded membranes was analyzed by peeling test (ASTM D1876) at Korea Polymer Testing & Research Institute in South Korea. The T-peel test was conducted using Universal Testing Machine 300 mm/min at a bonding temperature of the electrospun membrane: 45, 70, 100, 120 degrees of the bonding process.

### Microsphere perfusion

To confirm perfusion in the bonded electrospun membrane with a vascular network, 5wt % 10 µm Fluorescent beads (Thermo Fisher Scientific) in PBS solution was perfused by a syringe pump (New Era Pump System Inc., Farmingdale, NY, USA). The fluorescence of the perfused bead was observed with NIGHTSEA (NIGHTSEA, Lexington, MA, USA).

### Cell seeding and culture

The bonded embossed membranes with the vascular network were sterilized with EO gas, and then the membranes were coated with collagen (3 mg/ml, Thermo Fisher Scientific). To compare the cell adhesion on the coated surface, fibronectin (10 µg/ml, Thermo Fisher Scientific), gelatin (1% w/v, Sigma), or collagen (3 mg/ml) was coated in a hollow channel of the membranes. Red fluorescent protein (RFP) expressing human microvascular endothelial cells (R-HMVECs) (Olaf pharmaceuticals, Worcester, MA, USA) or R-HMVECS and mouse embryo fibroblasts cells (pericytes, 10T1/2, ATCC, Manassas, VA, USA) were used in this study. R-HMVEC cells were cultured in endothelial cell growth medium (EGM-2 MV, Lonza, Walkersville, MD, USA) supplemented with Antibiotic–Antimycotic solution (1% v/v, Thermo Fisher Scientific) and mouse embryo fibroblasts cells (10T1/2) were cultured in Dulbecco’s Modified Eagle Medium (DMEM, Thermo Fisher Scientific) supplemented with fetal bovine serum (10% v/v, FBS, Thermo Fisher Scientific) and Antibiotic–Antimycotic (1% v/v) solution at 37 °C in a 5% CO_2_ humidified atmosphere. The R-HMVECs cells were resuspended in gelatin (100 µl, 5%) solution at a density of 1 X 10^7^ cells/ml and inserted with 1 ml/min seeding velocity using a syringe pump into the bonded-electrospun membrane. The cell-seeded membranes were incubated at 37 °C in a 5% CO_2_ humidified overnight to permit cell adhesion. Following 18 h of incubation of membranes in a cell culture incubator, sequential seeding of R-HMVECs was carried out to line the cylindrical lumen by inverting the membrane. Mouse fibroblasts cells were seeded after R-HMVEC adhesion in 50 µm culture mediums at a density of 2 × 10^6^ cells/ml onto both outer surfaces of the membrane.

### Flow culture for vascularized membranes

The bonded embossed membranes with endothelial cells were cultured statically or with a gravity-driven flow system. The medium reservoir was connected with the embossed membranes through a channel having a 100 µm internal diameter and 4.5 cm length at the 14 cm height. The flow rate of the medium from the reservoir was set by adjusting the length and height of the channel so that it was calculated as 4 ml/day in advance. The flow rate of the culture medium was performed based on the equation [23] described in the results.

### SEM observation

The cell-seeded samples were fixed with glutaraldehyde (1% v/v, Sigma) and paraformaldehyde (1% v/v, PFA, Biosesang, Seongnam, South Korea) in PBS for 30 min at 4 °C. The samples were then fixed in osmium tetroxide (1% v/v, OsO_4_, Sigma) in dH_2_O for 30 min at RT. Dehydration of the fixed cells was performed using ethanol series for 20 min in each and the cells were substituted with ethanol. Samples were immersed overnight in pure hexamethyldisilazane (HMDS, Sigma) followed by air-drying in a fume hood. Samples were coated with Pt by sputtering. Observed with SEM system (Seron Technologies) at an accelerating voltage of 10 kV.

### Permeability analysis

Permeability was evaluated by measuring the diffusion of 70 kDa FITC-Dextran (Sigma) across the membrane of the Transwell [24] and vascularized channel. In this experiment, both systems of the vascularized membrane with linear channel and Transwell® inserts were used. The vascularized membranes have a linear channel for verifying definite effluence.

For the experiment, the vascular channel of the membrane without cell adhesion and with endothelial cells and pericytes on day 7 after cell seeding, and the vascularized membrane were submerged in the basal medium without growth factors and serum. Then, FITC-dextran solution (30 µl, 200 µg/ml) with the basal medium was perfused inside of the channel of the flexible membrane with 0.5 ml/min rate. And the fluorescence of the perfused FITC-Dextran was observed with NIGHTSEA.

In the Transwell experiment, the electrospun membrane attached Transwell inserts were placed in a 12-well plate. To confirm the permeability coefficient with various time points, 1.5 ml PBS solution was filled with Transwell bottom, and then FITC-Dextran solution (0.5 ml, 200 µg/ml) was added inside the insert. The three types of Transwell inserts with no cell, only endothelial cells, or co-culture of endothelial cells and pericytes between the membrane have been experimented with. The experiment was performed at a 37˚C incubator. The permeability was analyzed every hour for 4 h, and aliquots (100 μl) were taken from the bottom side of the well plate and place in a 96-well plate. The membrane permeability coefficient (*P*) was determined as the following equation:

1 $${\varvec{P}} = \frac{{{\varvec{dQ}}}}{{{\varvec{dt}}}} \times \frac{1}{{{\varvec{AC}}_{0} }} = \frac{{\left( {\frac{{\Delta {\varvec{C}}}}{{\Delta {\varvec{t}}}}} \right)}}{{{\varvec{C}}_{0} }} \times \frac{{\varvec{V}}}{{\varvec{A}}}$$

where P is the permeability coefficient, *dQ*/*dt* is the diffusion rate of FITC-dextran, A is the surface area of the insert well, and *C*_*0*_ is the initial concentration of the FITC-dextran solution in the insert well. The light transmittance of the outer soup was measured with a fluorometer (Victor 2, Perkin Elmer) within the wavelength 485–535 nm.

#### Cryosection for embossing membranes

The samples were harvested and fixed by incubation in PFA solution (4%) for 24 h, then kept in gelatin solution (7.5%) in sucrose solution (10%, Duchefa Biochemie, Haarlem, Netherlands) for 3 days at 37 °C. The samples were incubated in 4% PFA for 1 h, RT, and then transferred to an embedding mold containing optimum cutting temperature (OCT) cryostat embedding compound (Tissue-Tek, Torrance, CA, USA), then frozen in the cryostat chamber. The frozen blocks were sectioned 40 μm thick and dried on a 26 °C hot plate.

#### Immunofluorescence Staining

The cultured samples were harvested and fixed by incubation in 4% PFA and incubated for 1 h at 37 °C with primary antibodies: anti-CD31, anti-αSMA, and anti-Collagen I (Table S1). The samples were then further incubated for 1 h at room temperature with either Alexa Fluor 488 goat anti-mouse secondary antibodies (Thermo Fisher Scientific) (1:100). The F-actin of the vascularized membrane was visualized with a 1:40 dilution of Alexa Fluor 488 phalloidin (Thermo Fisher Scientific) and cell nuclei were counterstained using DAPI nucleic acid stain (Thermo Fisher Scientific) (Table S1). Sections were viewed using an LSM880 confocal microscope and images were captured and analyzed with digital software (Zen2011, Carl Zeiss, Oberkochen, Germany) and FIJI/ImageJ (National Institutes of Health, Bethesda, MD, USA) software. For analysis of the adhered cell alignment in the vascular network membranes, cell directionality was analyzed using the actin express figure after 7 days with the flow or static culture. Fast fourier transform (FFT) analysis, an indirect method used to examine endothelial cell alignment in the F-actin images, was used to evaluate the orientation of endothelial cells based on the generated vascular network channel in confocal images.

#### Gene expression analysis

The harvested samples were frozen on day seven for total RNA extraction. The extracted RNA was analyzed by EBiogen (Seoul, South Korea). The used genes for analysis were shear stress-related genes (Table S2).

#### Statistical analysis

All data are reported as the mean ± standard deviation (SD). Experimental groups were compared with ANOVA of Origin (Originlab Corporation, Northampton, MA, USA) and two-sample t-tests using Microsoft Excel 365 (Redmond, WA, USA), and *p* < 0.05 was considered statistically significant.

## Results

### Alginate vascular network mold

The alginate vascular-network mold for a vascularized membrane was cross-linked onto the contact surface of a gelatin gel containing CaCl_2_ solution using a master mold made in advance with PDMS, and then a vascular tree sacrificial mold was dried in the shape of the master mold. The alginate mold sizes are as indicated in the figure from the thick thickness to 1000, 551.72, 448.28, and 172.41 µm, and the mold diameter is about 300 µm (Fig. S2). The thickness of the fabricated alginate mold was reduced by about 50 ~ 70% compared to the thickness of the original master mold. It is inferred that after the alginate mold is crosslinked, the solvent that remained within the alginate vascular mold is vaporized during being dried away (data not shown). The fabricated alginate vascular mold was sandwiched and bonded between the surface-treated electrospun membranes, and then completely dissolved using 0.2 m trisodium citrate hydrate for 3 days (Fig. 1).

### Surface characterization

**Fig. 2:**
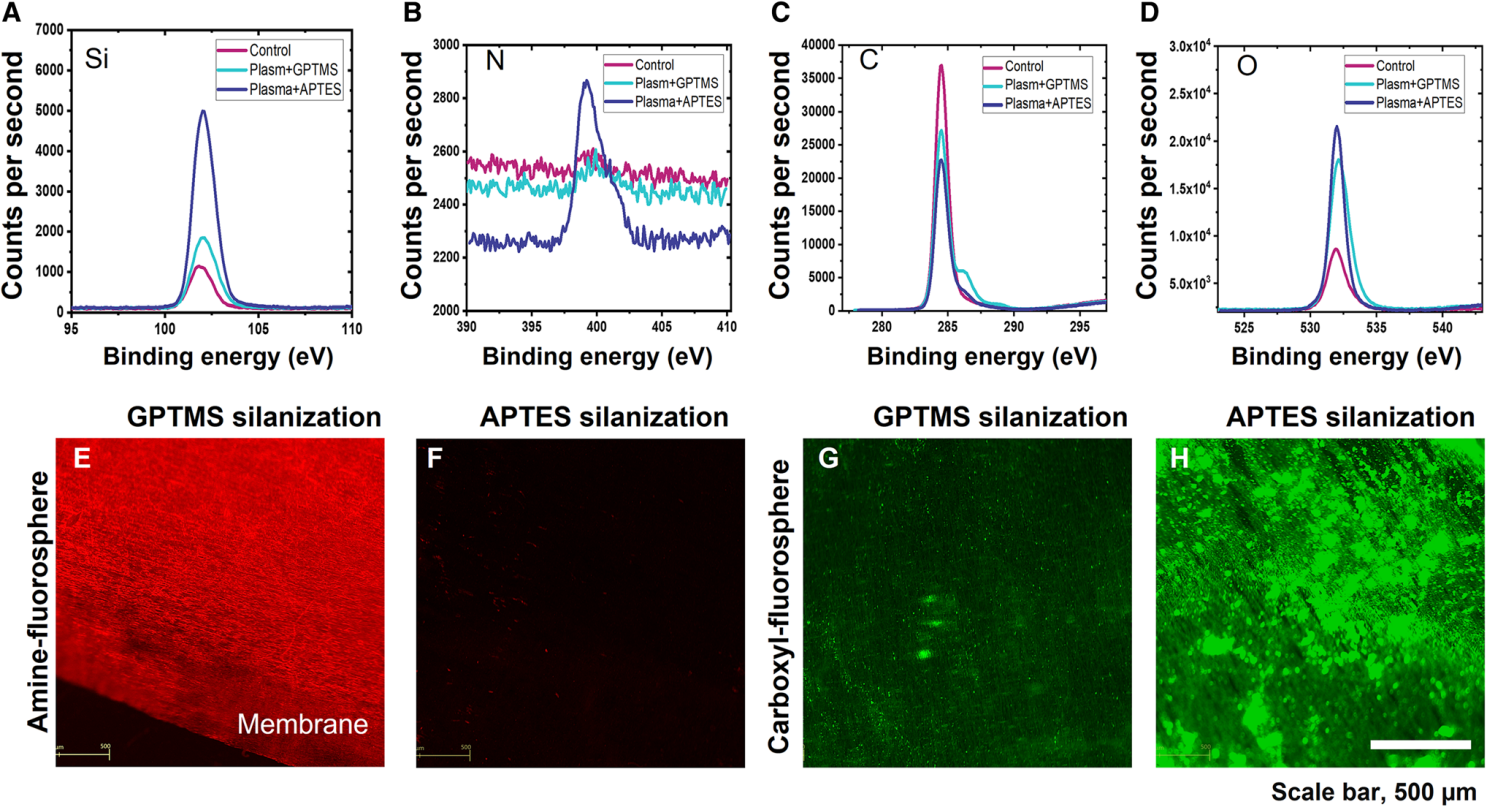
Characterization of the silanized surface on the membrane. XPS analyses: **A** Si2p, **B** N1s, **C** C1s, and **D** O1s peaks on the surfaces of control (no-treated), ATPES-treated, and GTPMS-treated sample. **E**, **F** Fluorescence measurements: amine-functionalized fluorospheres reacted on **E** GPTMS-treated and **F** ATPES-treated membranes. Carboxyl-functionalized fluorospheres reacted on **G** GPTMS-treated and **H** APTES-treated membranes. Scale bar is 500 µm

Figure 2A, B, C and D and supplementary fig. S3 show the results of XPS analyses on the silanized surface of the coverslip. In the APTES and GPTMS-treated samples, it can be confirmed that the intensity of oxygen (O) was increased by the condensation process of ethoxy (–C_2_H_5_O) and methoxy (–CH_3_O) groups with surface hydroxyl groups. In addition, it is shown that the intensity value of N increased on the APTES-treated surface due to the influence of the amine group. Figure 2E, F, G and H shows the results of fluorescence measurements made using functionalized fluorescence spheres. Figure 2E shows the binding of amine-functionalized fluorospheres (excitation 580 nm/emission 605 nm) in a GPTMS fixed membrane. As can be seen, bright red fluorescence appeared on the surface of the GPTMS fixed membrane. Amine-functionalized fluorospheres reacted on the APTES-fixed membrane surface. As shown in Fig. 2F, no red fluorescence was observed confirming that APTES was successfully immobilized on the membrane surface, and the red fluorescence in Fig. 2E is the result of specific adsorption of amine-functionalized fluorescent spheres on the GPTMS immobilized membrane surface. The green fluorescence in Fig. 2H shows the successful binding of carboxylate functionalized fluorescent spheres (excitation 505 nm/emission 515 nm) on the APTES-fixed membrane surface. Green fluorescence was also observed on the GPTMS-fixed membrane surface as the carboxylate groups can also react with GPTMS (Fig. 2G). Carboxylate-functionalized fluorospheres also reacted nonspecifically on the exposed membrane surface. However, the fluorescence was negligible.

The figures showed that after the bonding process, the PLCL electrospun membranes had nano-sized fibers (Supplementary Fig. S4). As the bonding temperature of the membranes is higher, 100 and 120 degrees, the nanofibers on the contact surface with the alginate vascular mold at the inner surface of the membrane were observed pressed wider compared to 45 and 70 degrees.

### Perfusion capabilities between the membrane

**Fig. 3:**
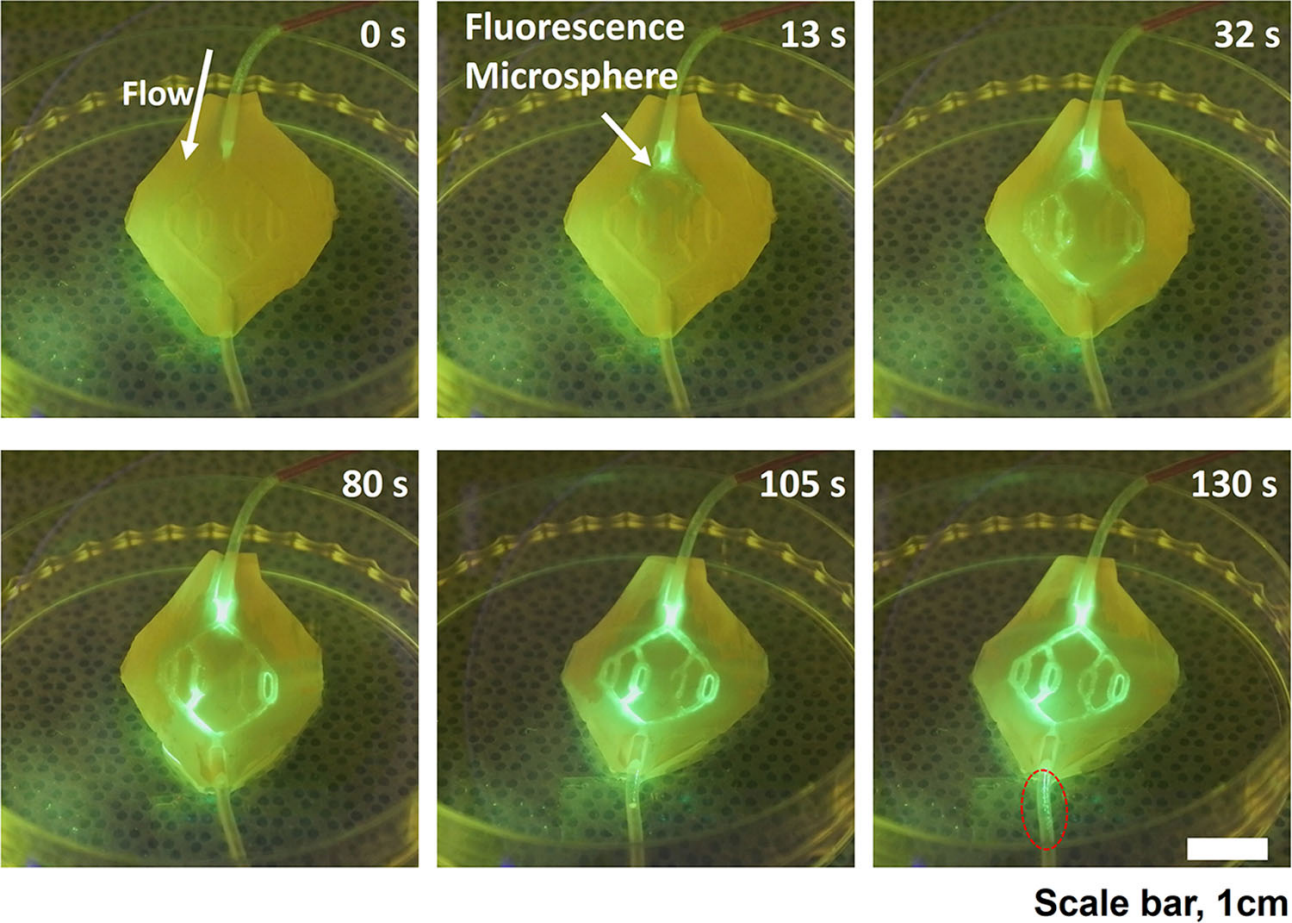
Video frames capturing fluorescent microsphere location within a bonded membrane over time. The fluorescent images of the vascular network were visualized with 5% (w/v) green-fluorescent microspheres (10 µm) solution during 1 ml/min perfusion. Scale bar is 1 cm

After the alginate vascular tree mold was dissolved, 5% (w/v) fluorescent microspheres were diluted in PBS solutions and flowed using a syringe pump in order to confirm that a perfusable ability (perfusability) and space formed between the membranes. As shown in Fig. 3, when injection of the fluorescent beads was performed using a syringe pump, the fluorescent substance could be observed flowing inside the membrane during the running of the syringe pump. Additionally, the adhesion strength of thin membranes was confirmed by the T-peel test (Supplementary Fig. S5). It is shown that the peel test results increase as the bonding temperature increases. Above 120 degrees, in our bonding process, it was not possible to progress of bonding for a long time owing to high temperature for home-made setting, and there was no difference in the peeling test results compared to the case of 100 degrees for 2 h. The subsequent cell seeding process and culture were performed with the sample bonded by applying 100 degrees for 2 h.

### Cell seeding and culture with vascularized membranes

**Fig. 4:**
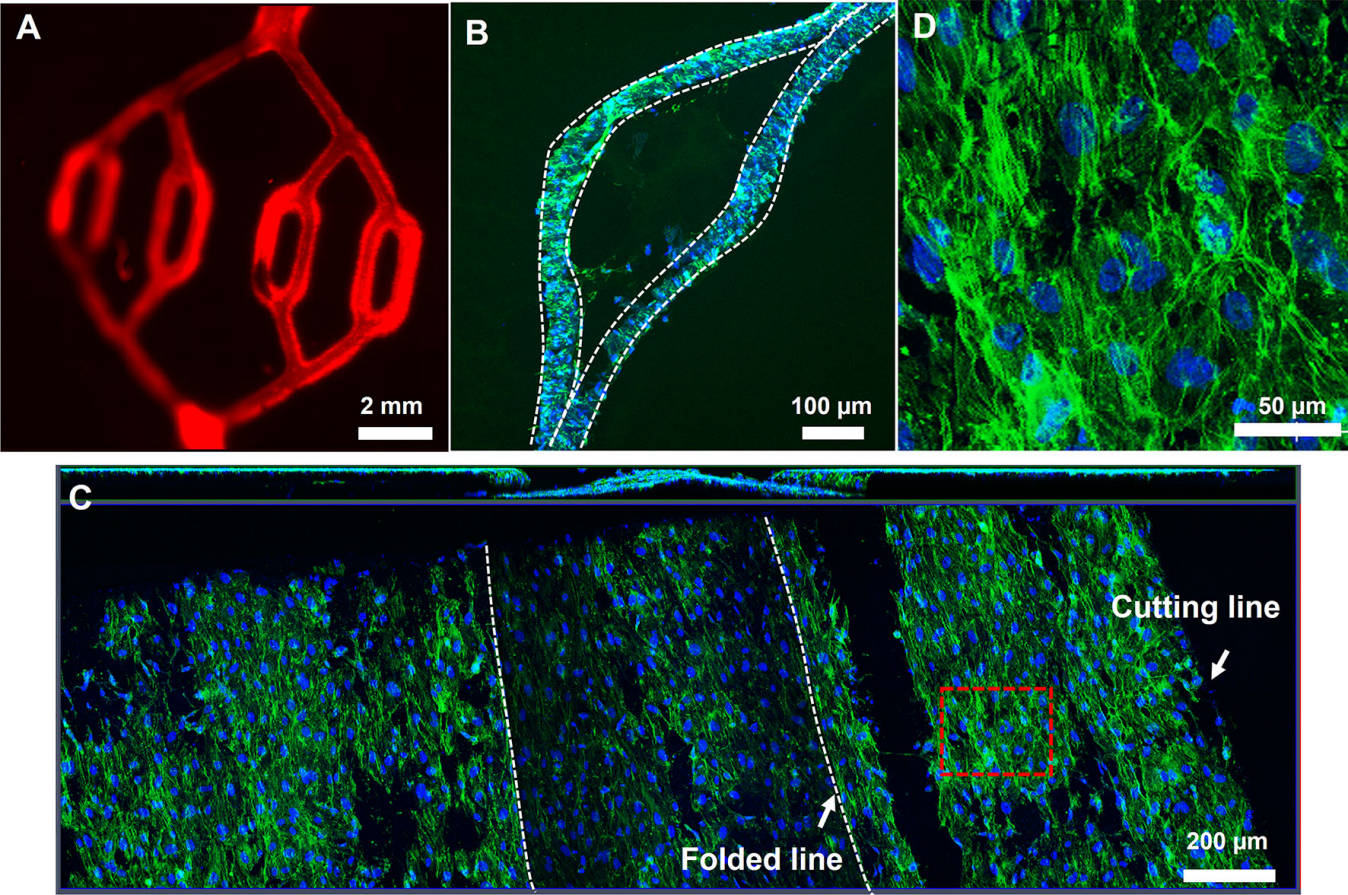
**A** *In vitro* cell-cultured vessel using with the bonded-electrospun membrane **B** cross-sectional images of actin staining. The dashed line shows the electrospun membranes. Scale bar is 100 µm. **C** The inside of the bonded electrospun membrane. The white dash lines indicate the folded line after cutting of the top of the embossed membrane. scale bar is 200 µm. **D** The enlarged region of the red dashed square in Fig. 4C. Scale bar is 50 µm

**Fig. 5:**
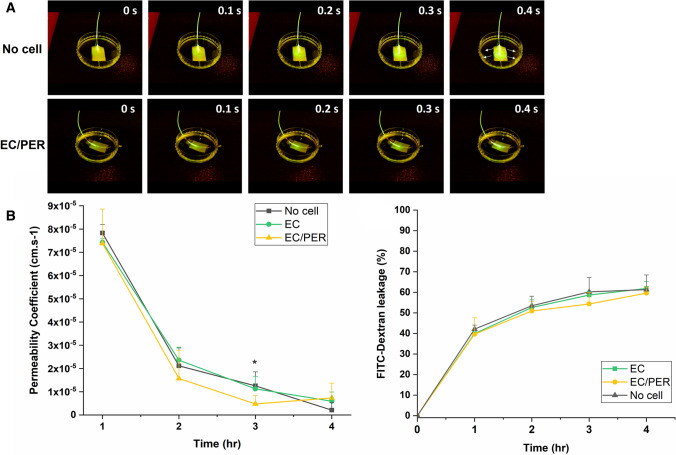
Permeability of the FITC 70 kDa dextran tracer through **A** electrospun vascularized membranes and **B** transwell inserts without cells and coated with cultures of endothelial cells or endothelial cells and pericytes after 7 days in static culture. Error bars represent the standard deviation of four independent experiments. *p < 0.05

To endothelialize the bonded membrane, human endothelial cells were seeded into the bonded membrane. As shown in the figures (Supplementary Fig. S6), the endothelial cells are all adhered at day 4 to the amine- or epoxy-treated membrane surface without additional surface coating. The SEM figures show that the endothelial cells in the membrane adhered to and grew along the nanofibers of the membrane at day 4. In order to increase endothelial cell proliferation, adhered endothelial cells were compared proliferation by different coating conditions such as 3 mg/ml collagen, 10 µg/ml fibronectin, and 1% gelatin at day 7 (Supplementary Fig. S7). It was confirmed that the endothelial cells onto the collagen-coated surface were expressed in staining of F-actin and VE-cadherin compared to other coating conditions. Therefore, all subsequent cell attachment was performed under collagen-coated conditions. After seeding and culturing the endothelial cells in the bonded membranes, the cross-section was confirmed that the cells were attached along with the shape of the bonded membrane at day 7 (Fig. 4A). The endothelial cells have adhered inside the membrane, but after 4 days, it can be confirmed that cells passed through the membrane and adhered to the outside as well. To check the cell adhesion inside of the bonded membrane, the membrane was cut at the top point of the embossed membrane with a vannas scissors (Fig. 4B). The endothelial cells were covered on the total area of the inside of the membrane. Also, the cells were covered with cell-junction inside of the membrane for the EC monolayer (Fig. 4C). As shown in the figure (Fig. 5A), in the sample with no attached cells, it can be confirmed that the FITC-dextran solution leaked out directly through the bonded membrane, but in the cell-cultured membrane, it was shown that the fluorescent solution flow along the path of the bonded membrane.

In the experiment of permeability analysis, Fig. 5B revealed that the permeability coefficient of the FITC 70 kDa Dextran tracer molecule has similar permeability in all samples with the Transwell insert platform. However, at the time point of 3 h, the permeability coefficient of co-culture of endothelial cell and pericyte (4.75 ± 3.6 × 10^–6^ cm/s) was lower compared to no cell (12.52 ± 6 × 10^–6^ cm/s) and only endothelial cell (11.25 ± 5.4 × 10^–6^ cm/s) significantly.

### Perfusion culture of vascularized membrane

In that flow culture, the medium flow rate of the pump was 3.74 ± 0.39 m/day in the connected pump by applying a range of 1–10 dyn/cm^2^ of shear stress of veins. The calculation method used at this time is shown in equation (1) [25].

**Fig. 6:**
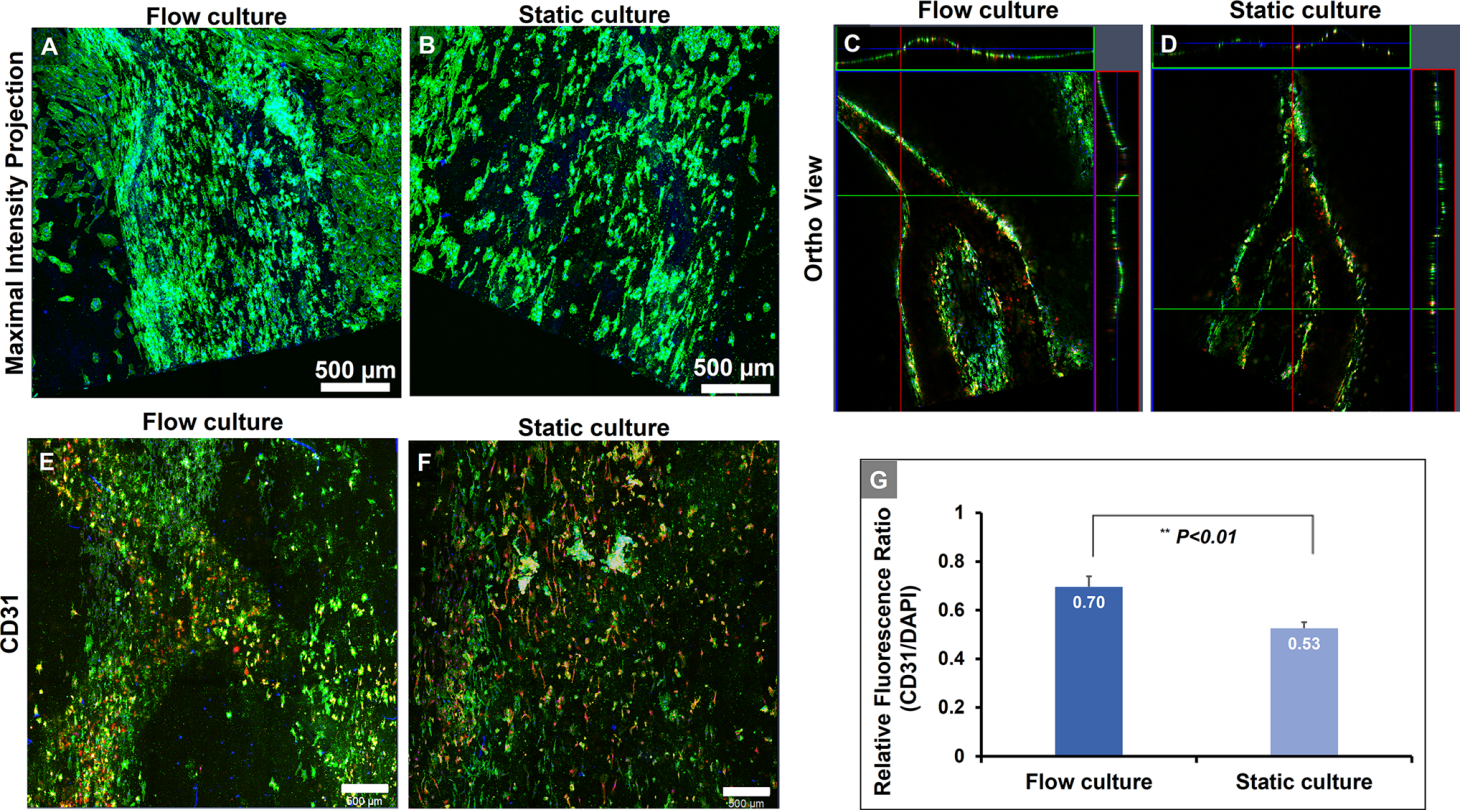
Flow culture improved endothelial cells proliferation in the engineered vascular network. **A**, **B** Representative maximum intensity projection of Z-stack, immunofluorescence images of cytoskeleton staining. **A** Flow cultured and **B** static cultured vascular network. Cytoskeleton: F-actin (green). Nuclei: DAPI (Blue). Scale bars represent 500 µm. **C**, **D** Ortho view of cytoskeleton staining. **C** Flow cultured and **D** static cultured vascular network. Cytoskeleton: F-actin (green). Endothelial cells (Red). Nuclei: DAPI (Blue). **E**, **F** Representative maximum intensity projection of Z-stack, Immunofluorescent images of CD31 staining. **E** Flow cultured and **F** static cultured vascular network for CD31 (Green), EC (Red), and DAPI for nuclear counterstain (blue). **G** CD31 ratio normalized per DAPI stained cells. The CD31 staining ratio is significantly increase compared with cells incubated under static conditions (**p < 0.01 vs. flow)

Endothelial cells were statically cultured for 3 days and then the cell-cultured vessel network was incubated with the connected pump for another 7 days. Endothelial cells were observed from the outside surface of the membrane after 7 days of *in vitro* culture. The seeded endothelial cells adhered along with the nanofibers of the membrane, and then it can be observed that the cell shapes are aligned along the embossed shape and flow direction at day 7 after pump connection. In the condition of static culture, it is shown that it is attached along with the nanofibers of the membrane, and the number of attached cells is less than that of the flow culture (Fig. 6A, B). In the ortho view of the vessel network sample, it can be shown that the endothelial cells adhere along the embossed surface of the polymeric membrane (Fig. 6C, D). Measuring the degree of alignment of vascular cells within the vascular network channel showed that the frequency of the directionality in the flow-cultured endothelial cells is higher at 4.20 degrees to the direction of the vascular channel within 7 days. The directionality of the vascular cells in the static condition showed highest amount at − 44.80 degree. The results showed that the adhered cells in static culture condition were aligned along the nanofibers of the membrane, independent of the flow channel. However, the vascular cells in the flow cultured sample were aligned highly along the channel direction. (Supplementary Fig. S8). In the condition of perfusion culture, it can be more clearly confirmed that more cells are observed along with the cross-section. As a result of staining CD31 at day 7 after perfusion culture, in the case of perfusion culture, it can be confirmed that the stained area was expressed along with the embossed shape of the membrane, but in the case of static culture, the CD31 expression is not clearly distinguished and it can be confirmed that it is widely spread. Additionally, the CD31 expression ratio was also increased in the perfusion condition (Fig. 6E, F and G).

**Fig. 7:**
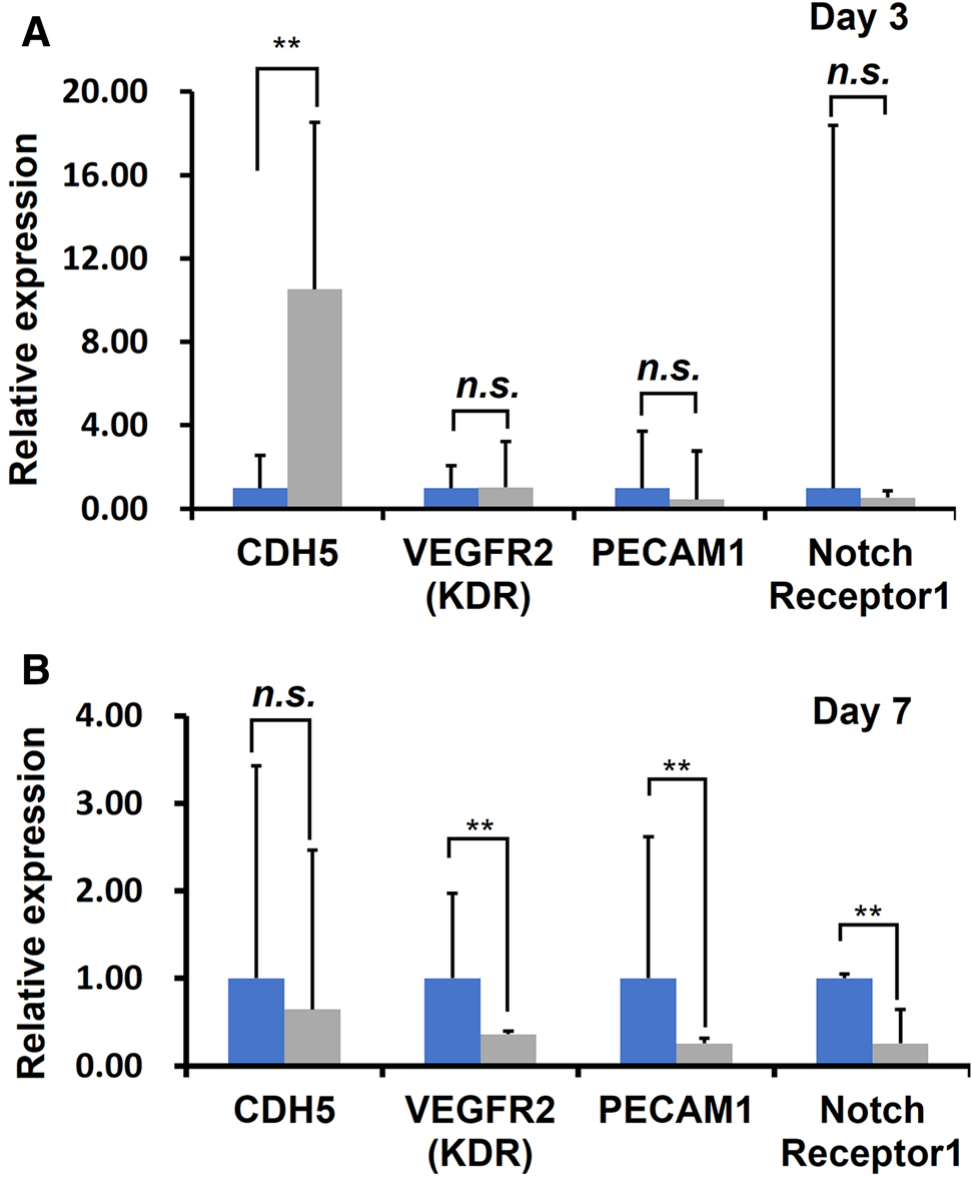
Gene expression of endothelial cells at **A** 3 and **B** 7 day of static (blue bars) vs. perfusion (gray bars) culture in the vascular network membranes. Error bars represent the standard deviation calculated from three independent experiments. ** indicates *p* < 0.01

The gene expression analysis of the vascular network after 3 and 7 days of perfusion culture revealed that CDH5 was upregulated to 10.53 times compared to those of static culture at day 3 (Fig. 7). However, after 7 days of perfusion culture VEGFR2 (KDR), PECAM1, and Notch Receptor 1 were downregulated to 0.36, 0.26, and 0.26 times compared to non-perfused culture.

For Vascularized complex tissue, endothelial cells were attached in the network shape between bonded electrospinning membranes, and then pericytes were seeded onto the outside of the vascular network after 1 day. After 7 days of culture, the fibroblast marker aSMA was expressed in the pericyte seeded samples (Supplementary Fig. S8). The expression of COL1 was not shown in the only EC-attached sample, but in the sample with both endothelial cells and pericytes, it was confirmed that COL1 was co-localized along the embossed surface, where the endothelial cells attached.

## Discussion

In this experiment, the electrospun membrane with the vascular network was cultured with perfusion for the maturation of endothelial cells *in vitro*. Electrospun membranes generally have more strength compared to hydrogel, and the degradation ratio of the membrane could have controllable properties. Also, the electrospun nanofibrous membrane could have a high surface area, a high surface area-to-pore volume ratio, high pore interconnectivity by nanofibers [26], and have flexible property by the addition of flexible material [27]. The vascular extracellular matrix is the elastic fiber network organized by medial smooth muscle [28]. Electrospinning technique could make it possible to produce fibrous matrix to mimic biological tissues, and the introduction of extracellular matrix proteins (collagen, fibronectin, and laminin) into the fibers makes these synthetic 3D matrices biocompatible [29]. Also, electrospun containing materials, PLCL have been shown to have good mechanical strength and tunable biodegradability[30]. We considered to application of skin or connective tissue, and PLCL material is experimented for 3D vascular networks membrane.

In this study, the bonding of two electrospun membranes is first tried for tissue engineering as far as we know. The bonding process can be challenging due to difficulties that arise when binding two non-uniform, polymeric, fibrous and porous membranes. When the contact surface is uneven for the bonding process, the contact points are less compared to the bonding of even planes for physical bonding, so bonding of electrospun membranes may not be easy. The proposed process using with chemical and thermal bonding was sufficiently robust to conduct perfusion culture with electrospun membranes for 7 days.

In the previous study, perfusion culture was conducted with cells on the single electrospun membrane [31–33]. However, electrospun membranes have not been rarely reported as the matrix for the three-dimensional perfusable vessel network *in vitro* because of their thinness and flexibility. In our study, the fluorescent beads were passed through the pre-embossed vessel network, and perfusion flow through the embossed vessel network was maintained. However, the electrospun membrane was not sealed against the liquid owing to its porosity and hydrophilicity. The 10 µm fluorescent beads or injected cells were passed through the vascular network of the electrospun membranes.

In the fabrication process of alginate vascular mold, the size and shape of the mold can be modified unlimitedly, and it can be manufactured by accurately implementing the vascular tree shape. Gelatin hydrogel was used for Ca^2+^ source for alginate crosslinking slowly. Polymerization of alginate was proceeded in the presence of Ca^2+^ diffused from gelatin hydrogel [34]. In this way of generating alginate sacrifice mold, it was possible to manufacture several alginate molds of the same shape at the same time.

In our experiment, vascular endothelial cells were seeded after mixing with a gelatin solution and then cultured while attached for endothelial cells to an electrospun membrane. The seeding with gelatin solution was used in order to more evenly adhere to the space inside the membrane. When endothelial cell seeding was tested using silicon tubing for confirmation of cell adherence, in advance, it was observed that cells adhered to the inside of the tubing more evenly than the ones adhered to using only a general medium (Supplementary Fig. S9). This was because the viscosity of the gelatin solution was higher than that of the medium, so that the cells were not concentrated to the bottom of the silicone tubing faster.

After the perfusion culture, the cultured vascular network was conducted by gene analysis using TRIzol RNA extraction method after removing the silicone tubing of the embossed sample. Shear stress-related genes such as notch1[35], VE-cadherin[36], VEGFR[37], and PECAM1[38, 39] were expressed during time points differently. In our system, shear stress was in the range 1–1.5 dyn/cm^2^, typical for venous vasculature. On day 3, CDH5 (VE-cadherin) was upregulated compared to that of the static group in the vascular network under perfusion culture, however, on day 7, PECAM1, VEGFR, and Notch1 were downregulated when compared with static conditions. These results showed that the shear stress-related genes were expressed differently at various time points. Some reports showed that flow shear stress differentially regulates endothelial cell adhesion molecules including VE-cadherin, notch1 cleavage, and platelet-endothelial cell adhesion molecule-1 (PECAM-1 or CD31) [36, 40, 41].

**Fig. 8:**
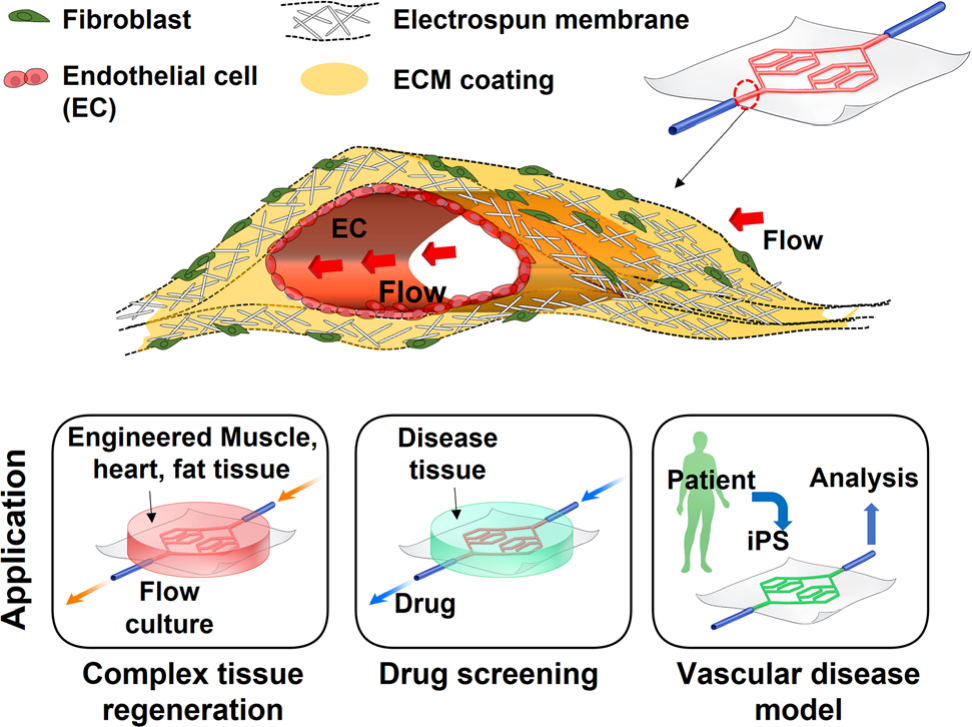
Application of Vascular network membrane for tissue engineering, drug screening, and disease model

In our experiment, collagen type I was coated to support endothelial cell adherence and proliferation. In further studies, vessel ECM such as laminin, fibronectin, and fibrillar collagens [42] can be used to mimic the vascular structure. In addition, fibroblasts were cultured on the outer surface of the vascular network with the electrospun membrane (Fig. 8). The generated vascular network could be mimic the natural vascularized tissue. Also, we successfully examined vasculogenesis *in vivo* experiments, in a previous study. The developed vascular network shows that the possibilities of vascularized modules for vascularized tissue both *in vitro* and *in vivo*.

The alone generated vascular network can be used as a vascularized membrane for skin necrosis and ischemic areas in our body. Also, the generated structure can be applied as a vascular tissue module for assembling with another tissue module (for example heart, muscle, fat tissues) to constitute a complex vascular tissue regeneration. Also, another disease tissue can be combined with the vascular tissue module, and the generated complex tissue was analyzed for drug screening. Additionally, vascular disease modeling was available using a patient’s fibroblast (induced pluripotent stem cell, iPS), and the vascular network was utilized as for vascular disease model.

In this study, we described for the first time perfusable *in vitro* engineered vascular network with electrospun membrane. Our engineered vascular network would represent an optimal model for investigating fundamental tissue/organ regeneration module, revealing complex interactions between the vascular network and other tissue/organ. In addition, the engineered vascular network is also capable of perfusion culture, so it can be cultured for a long time in the future and is expected to be used as a platform for drug screening.

## Supplementary Information

Below is the link to the electronic supplementary material.

Supplementary file1 (PDF 8760 kb)
