# Supplementary material for: Bonding of Flexible Membranes for Perfusable Vascularized Networks Patch
Source: Tissue Eng Regen Med. 2021 Dec 6;19(2):363–75. doi: 10.1007/s13770-021-00409-1 (PMC8971335; doi:10.1007/s13770-021-00409-1)
Supplement: Supplementary file 1 — Supplementary file1 (PDF 8760 kb) [file 13770_2021_409_MOESM1_ESM.pdf]

Supporting Information

Bonding of flexible membranes for perfusable vascularized networks patch

Soyoung Hong, Yejin Song, Jaesoon Choi, and Changmo Hwang**

Contents:

Figure S1. (A) Vascular network design for alginate mold and (B) the fabricated SU-8 mold

Figure S2. Fabrication process of alginate vascular mold.

Table S1. Materials used for immunofluorescence staining.

Table S2. RNA sequences for rt-PCR.

Figure S3. XPS Spectra of the silanized surface.

Figure S4. SEM images after bonding process at various bonding temperature, 45, 70, 100 and 120 degrees.

Figure S5. Variation of peel strength (T-peel test) with amine-epoxy-based adhesive at various bonding temperature.

Figure S6. Endothelial cells on day 4 onto the surface of APTES- or GPTMS treated surface.

Figure S7. Immunofluorescence staining of endothelial cells on the different coating conditions.

Equation S1. Shear stress equation

Figure S8. Orientation of adhered cells within the vascular network channel of the membrane subjected to flow or static culture.

Figure S9. Representative images of immunofluorescent staining after co-culture network on day 7.

Figure S10. Representative images of silicone tubing after endothelial cells injection with (A) gelatin solution or (B) culture medium

Figure S1. (A) Vascular network design for alginate mold and (B) the fabricated SU-8 mold

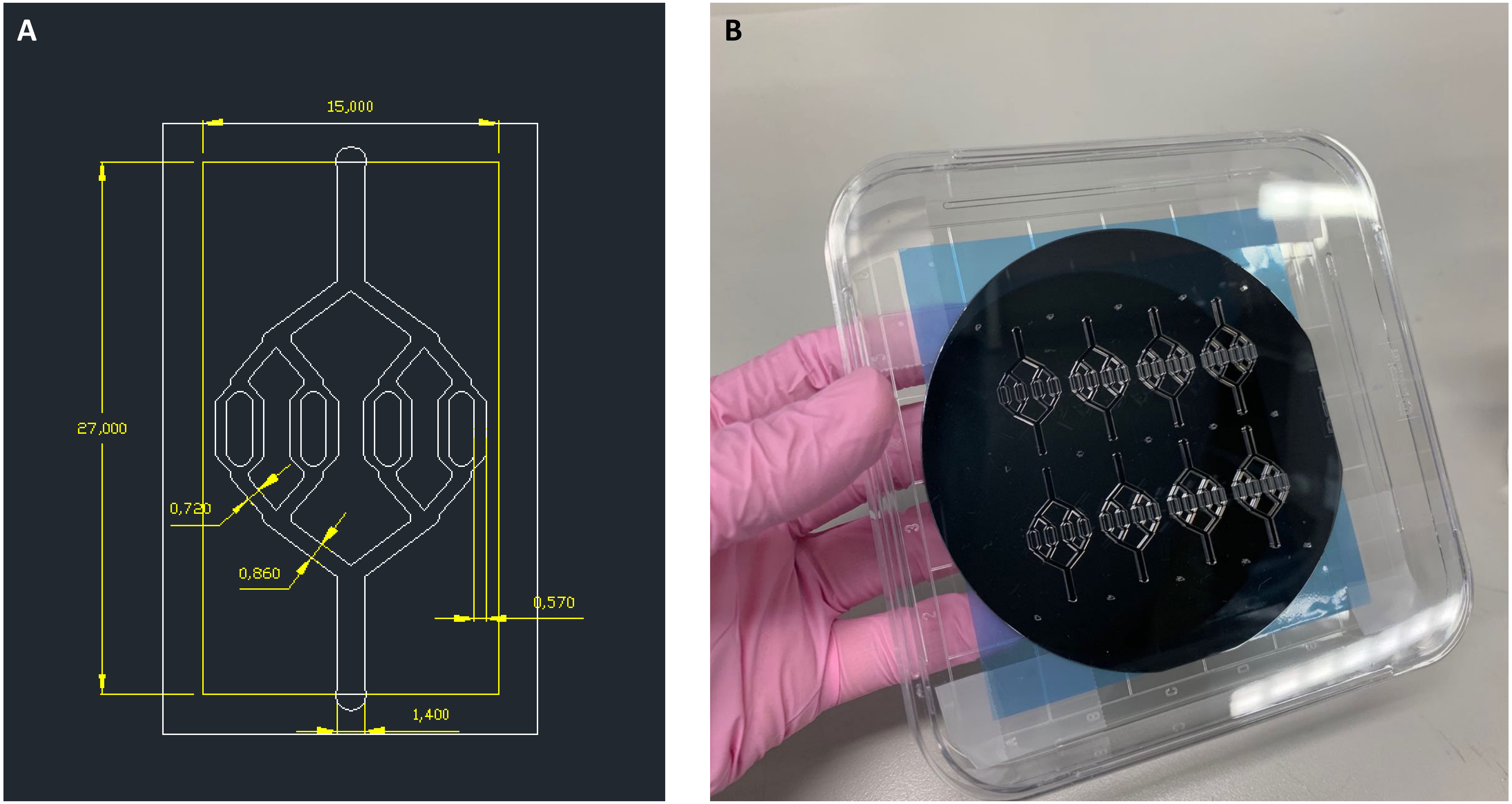

Figure S2. Fabrication process of alginate vascular mold

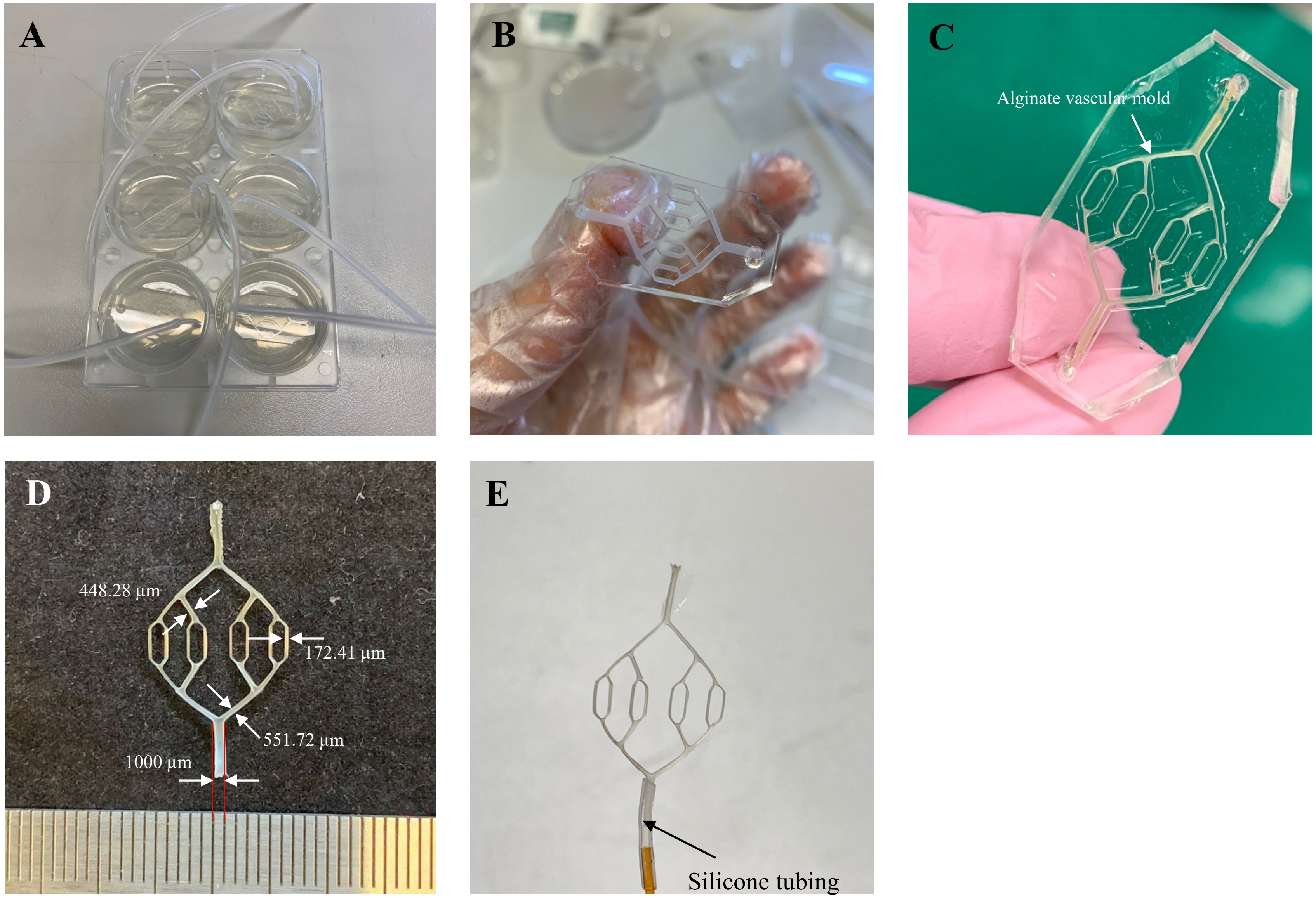

a) 6% alginate solution injection within the PDMS mold
b) Crosslinked alginate mold in the PDMS mold
c) After drying of alginate vascular tree mold
d) Alginate vascular mold
e) Insertion in silicone tubing

Table S1. Materials used for immunofluorescence staining.

Name	Type	Dilution	Company, No.	Annotation
**Anti-CD31**	mono	1:100	Thermo<br>MA3100	Endothelial cell marker
**Anti-Collagen I**	Poly	1:100	Abcam<br>ab34710	ECM
**αSMA**	mono	1:100	Thermo<br>MA5-11547	Smooth muscle cell marker
**Phalloidin**	-	1:40	Thermo<br>R415	F-actin
**4',6-Diamidino-2-Phenylindole, Dihydrochloride**		1:100	Thermo<br>D1306	DAPI
**Alexa Fluor 488 goat anti-Mouse**		1:100	Thermo<br>A11001	2nd antibody

Table S2. RNA sequences for rt-PCR.

Name	Accession Number	Sequence
**CDH5 (VE-cadherin)**	NM_001795.5	Forward: 5'-TTCACCTTCTGCGAGGATAT-3'<br>Reverse: 5'-TGATGGTGAGGATGCAGAGT-3'
**VEGFR (KDR)**	NM_002253.3	Forward: 5'-CCGGCCTGTGAGTGTAAAAA-3'<br>Reverse: 5'-GTCCGTCTGGTTGTCATCTG-3'
**PECAM1**	NM_000442.5	Forward: 5'-TCCCCAGAAGCAAAATACTG-3'<br>Reverse: 5'-TCCGATGATAACCACTGCAA-3'
**Notch receptor1**	NM_017617.5	Forward: 5'-CGACCTCCCCAACACCTACA-3'<br>Reverse: 5'-TGCAGTCGTCCACGTTGATC-3'

Figure S3. XPS Spectra of the silanized surface. Element peaks detected on the surface of the no treated, GPTMS, and APTES silanized surface.

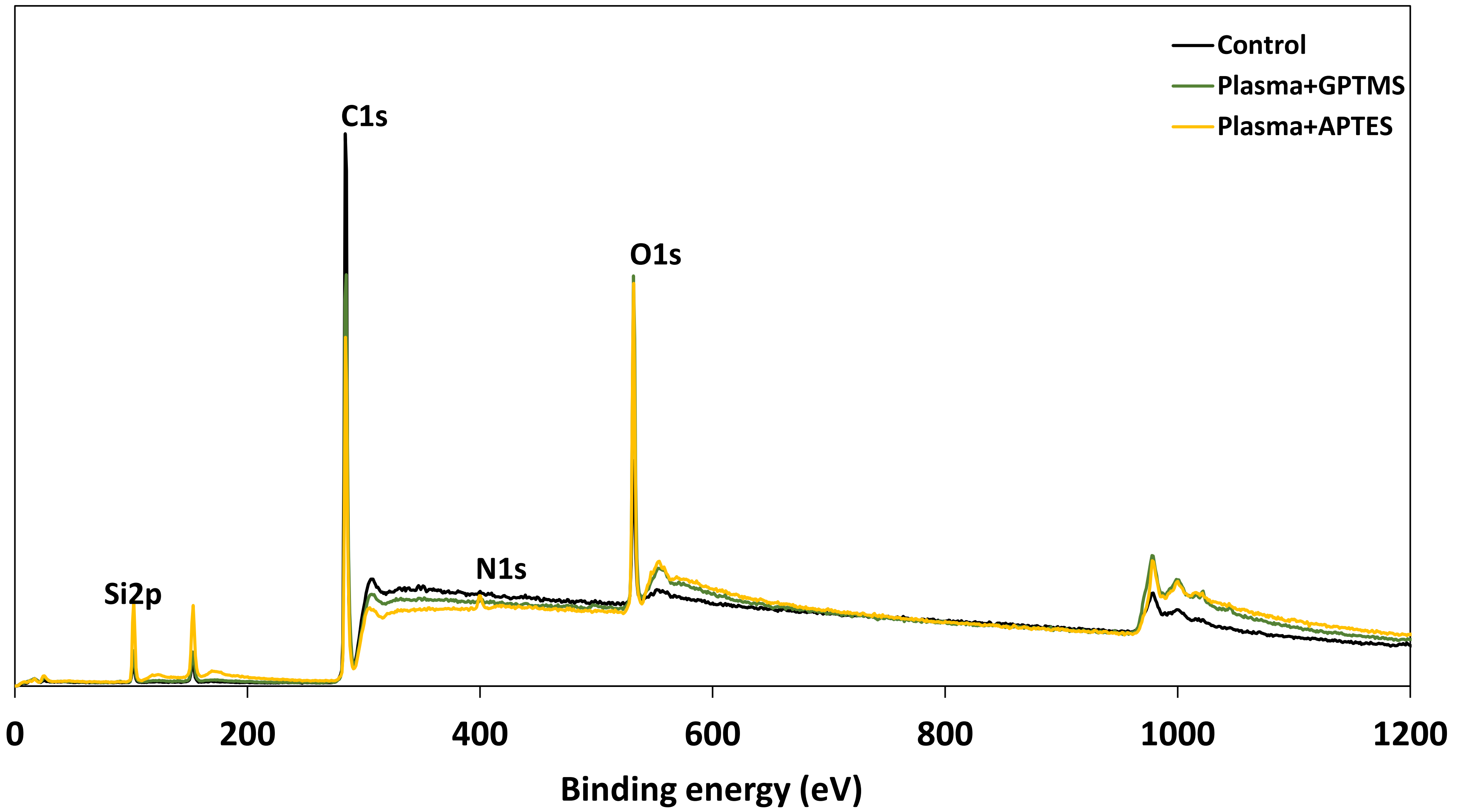

Figure S4. SEM images after the bonding process

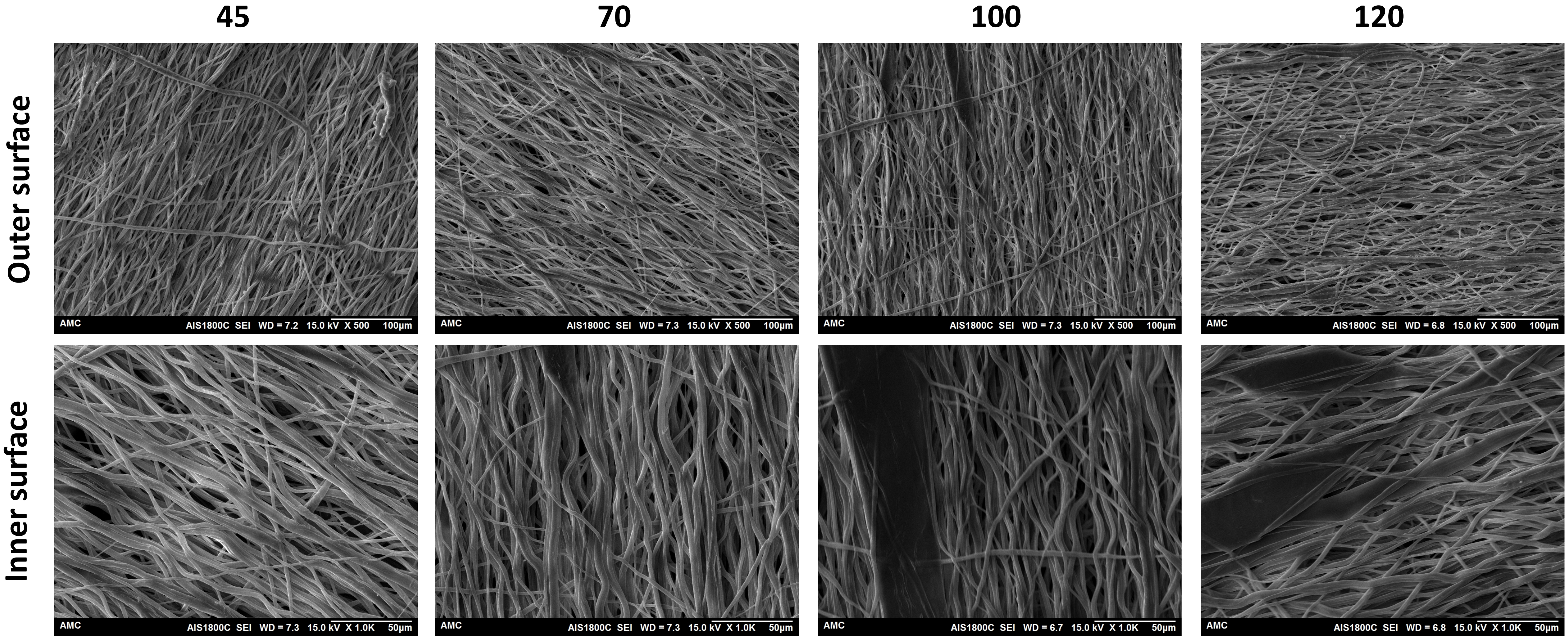

SEM observation of the membranes after the bonding process at various bonding temperatures, 45, 70, 100, and 120 degrees.

Figure S5. Variation of peel strength (T-peel test) with amine-epoxy-based adhesive at the various bonding temperature

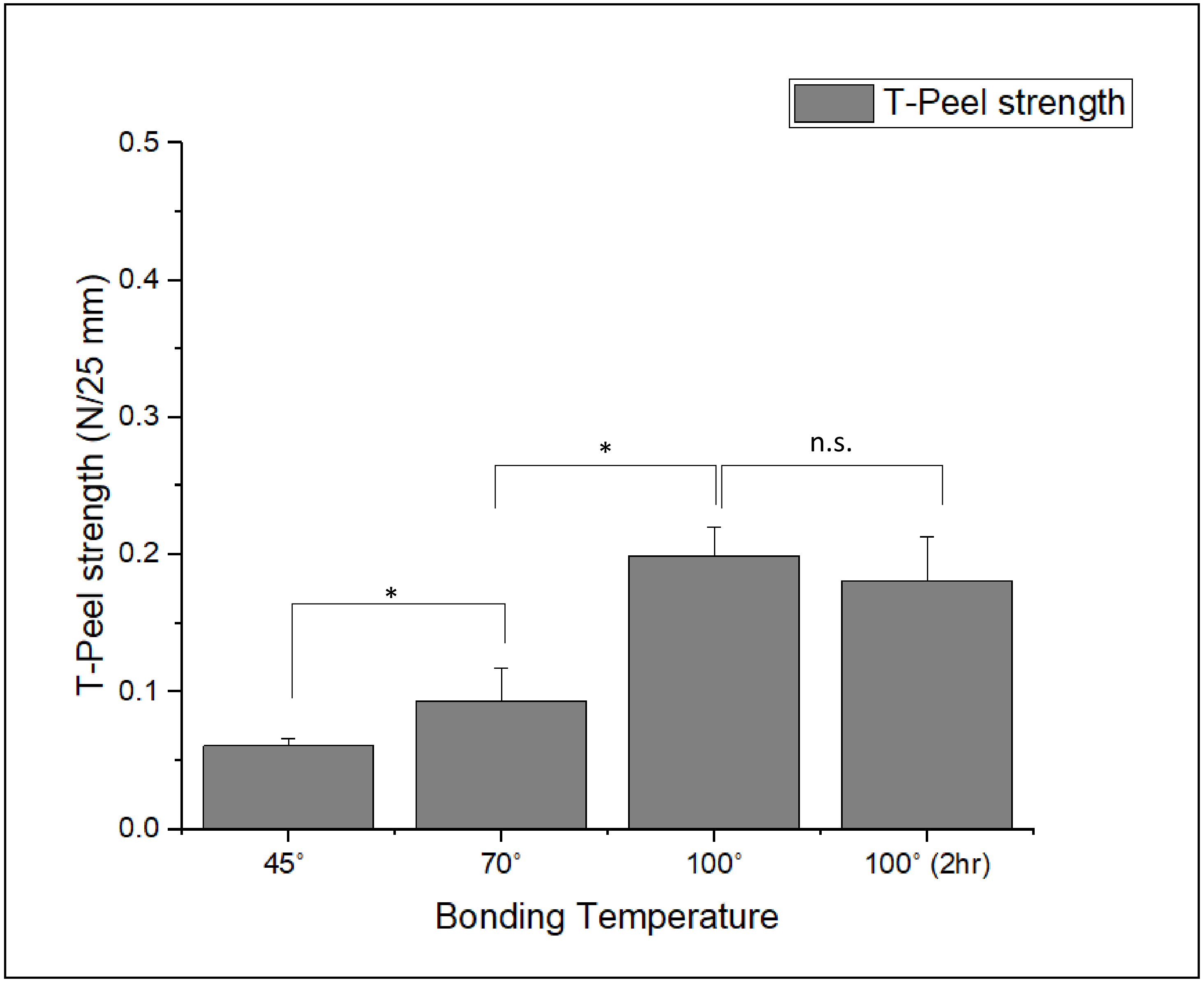

Figure S6. Endothelial cells on day 4 onto the surface of APTES- or GPTMS treated surface.

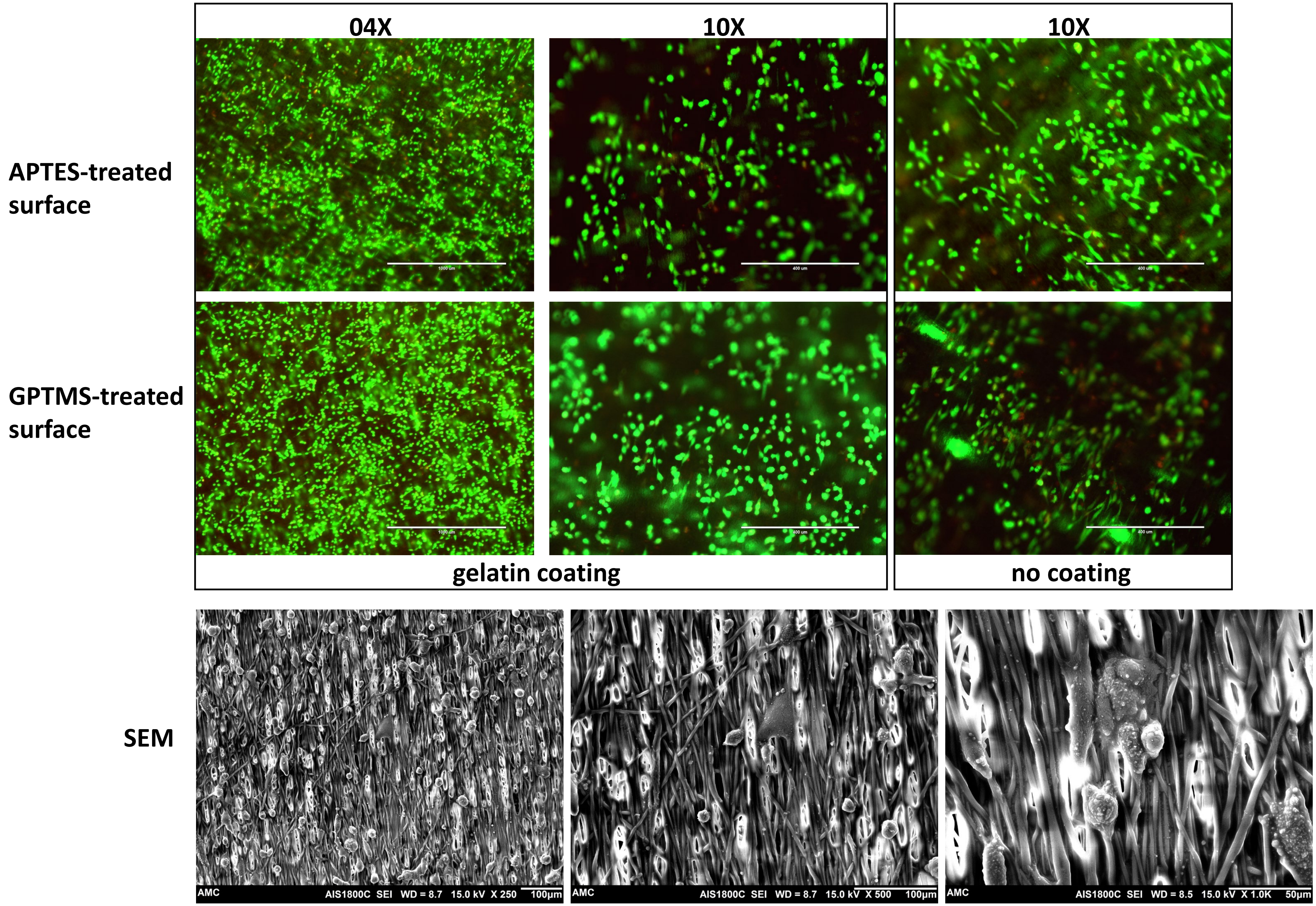

Endothelial cells were conducted LIVE/DEAD assay on day 4 onto the surface of APTES- or GPTMS treated surface. SEM images of endothelial cells and electrospun membrane.

Figure S7. Immunofluorescence staining of endothelial cells on the different coating conditions

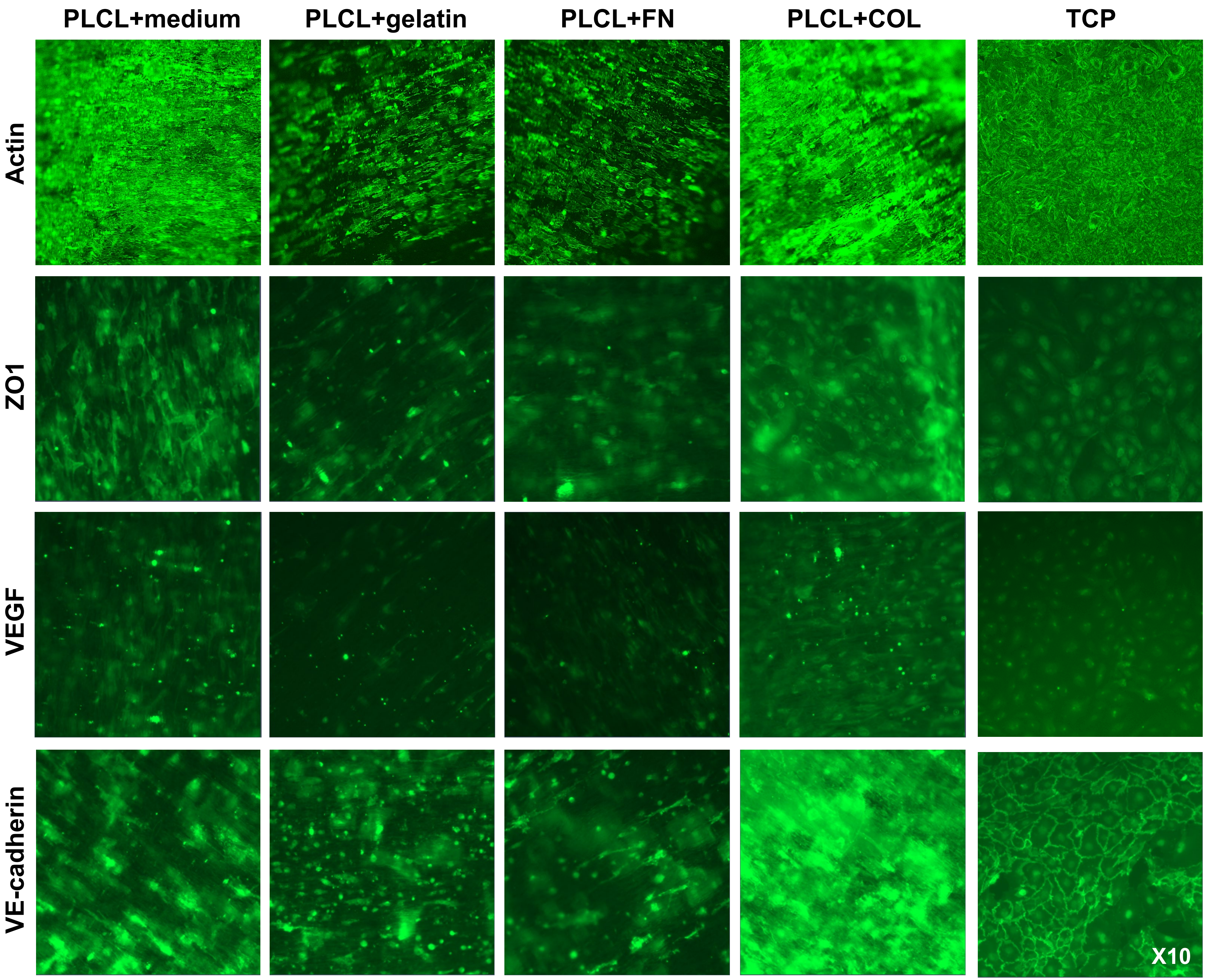

Adhered endothelial cells were compared proliferation by different coating conditions such as 3 mg/ml collagen (PLCL+COL), 10 μg/ml fibronectin (PLCL+FN), and 1% gelatin (PLCL+gelatin), pre-incubated with culture medium (PLCL+medium), and tissue culture plate (TCP) on day 7 for actin, ZO1, VEGF, and VE-cadherin.

Equation S1. Shear stress equation

$$\tau = \frac{6\mu Q}{wh^2}$$

$$\tau = vein' shear\ stress, 1 - 1.5\ dyn/cm^2$$

$$\mu = medium\ viscosity, 0.8cp$$

$$w = width, 0.05cm$$

$$h = height, 0.05cm$$

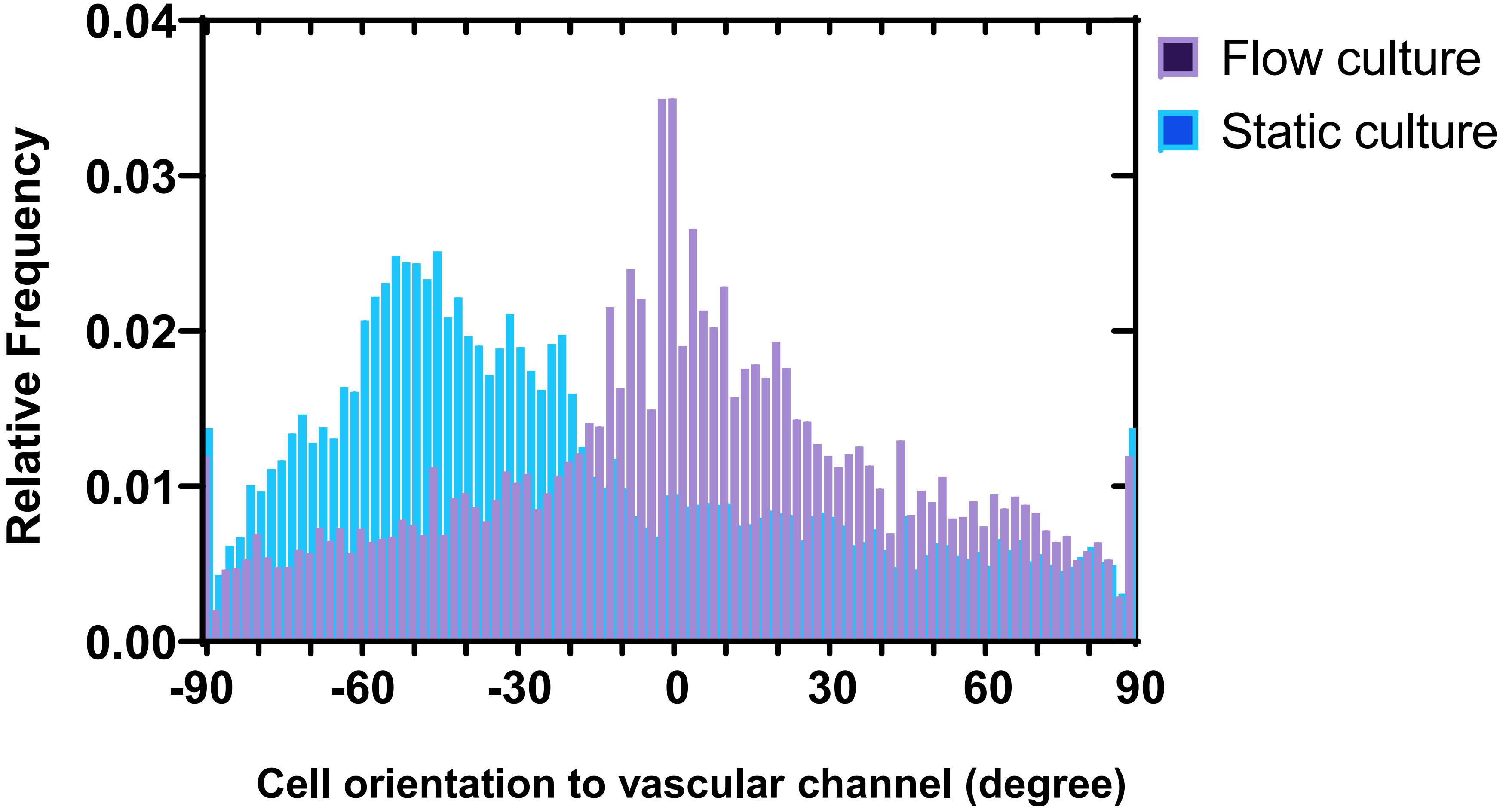

Figure S8. Orientation of adhered cells within the vascular network channel of the membrane subjected to flow or static culture. The direction of the generated vascular channel is shown 0 degree in this histogram. Cell alignment with the vascular channel was shown the intensity of the corresponding FFT signal analyzed using ImageJ software.

Figure S9. Representative images of immunofluorescent staining after co-culture network on day 7

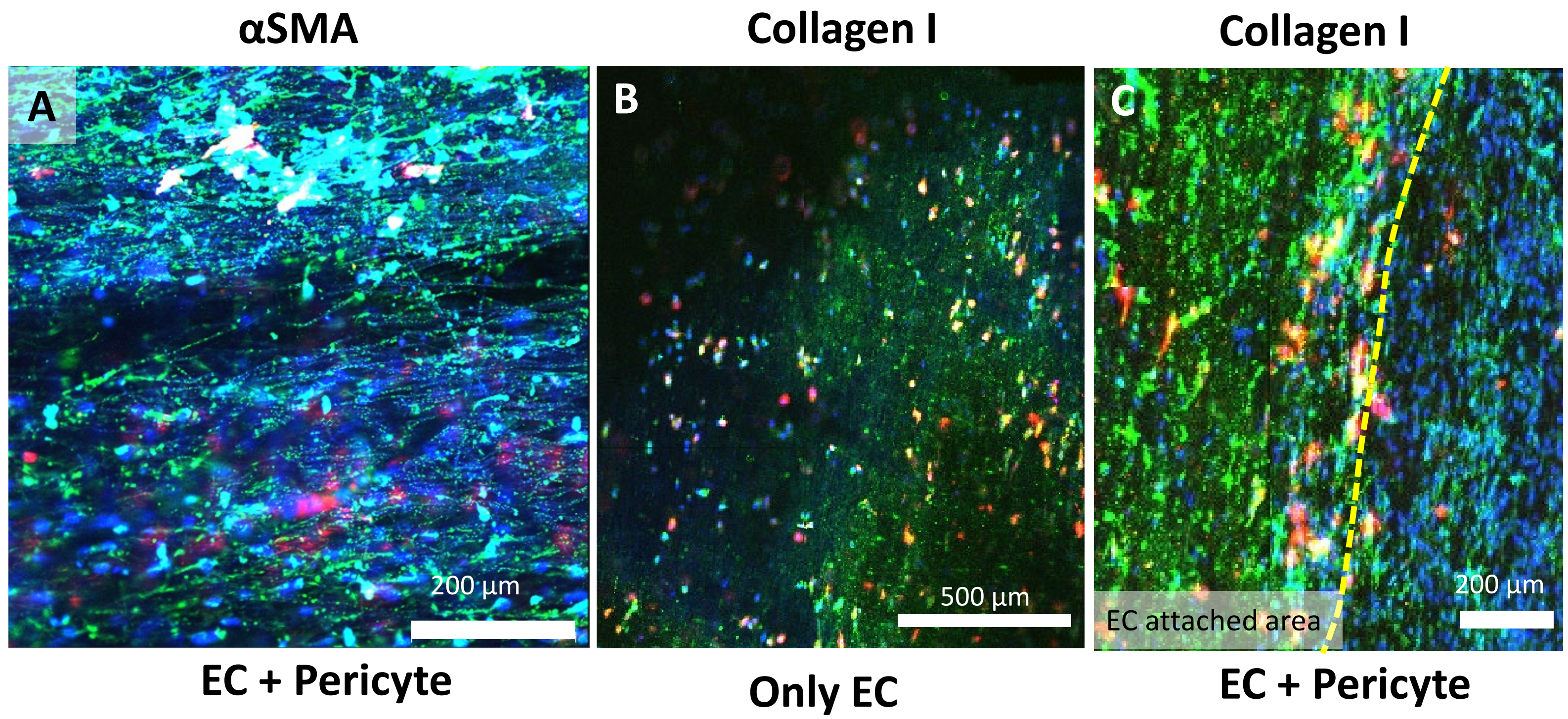

Endothelial cells and pericytes improved αSMA and Collagen type I expression on the outer surface of the engineered vascular network. (A) Representative immunofluorescence image of a co-culture network on day 7 for αSMA (green), EC (Red), and DAPI for nuclear counterstain (blue). (B) Representative immunofluorescence image of only EC culture network (B) and co-culture vascular network (B) on day 7 for Collagen type I (Green), EC (Red), and DAPI for nuclear counterstain (blue).

Figure S10. Representative images of silicone tubing after endothelial cells

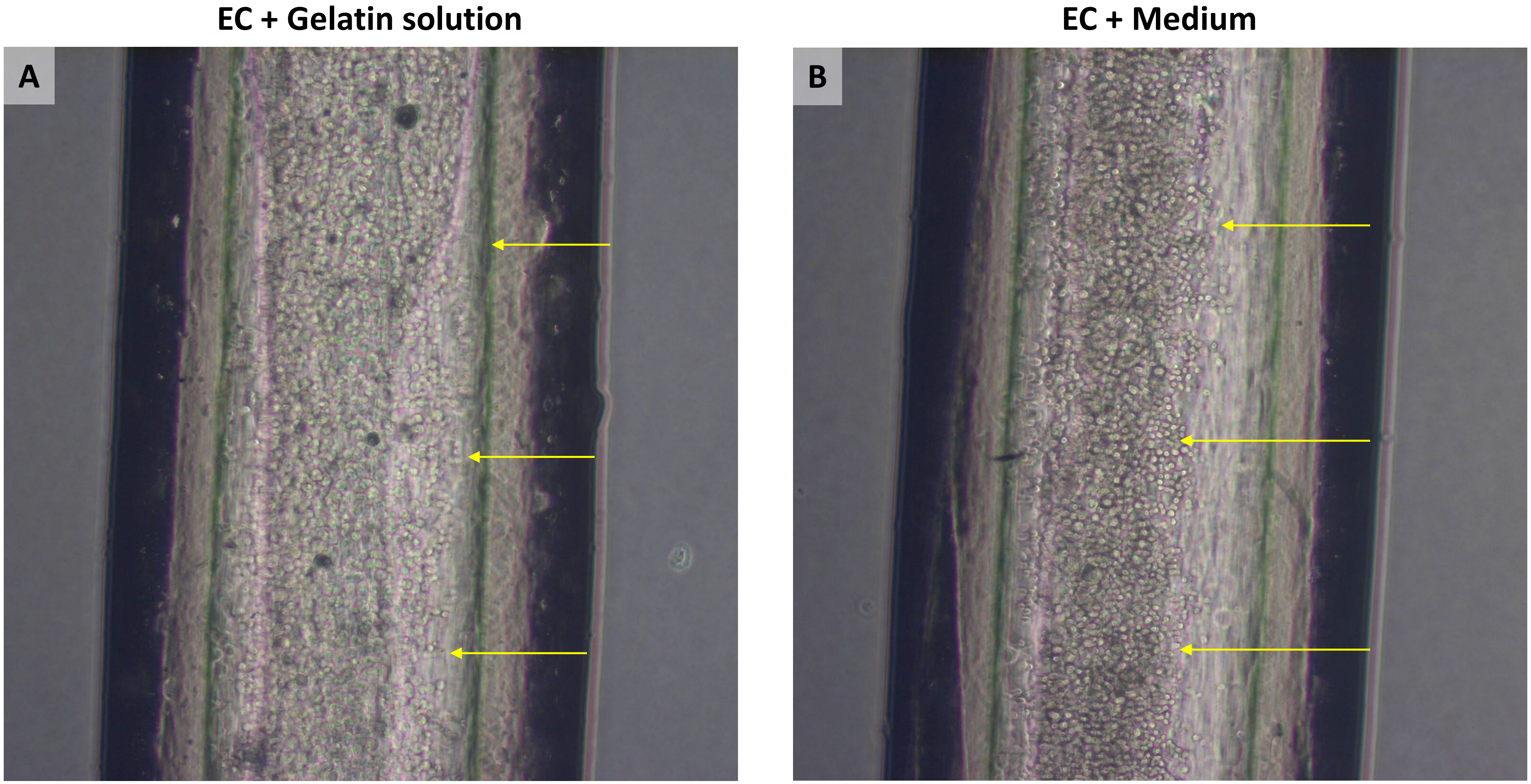

Representative images of silicone tubing after endothelial cells injection with (A) gelatin solution or (B) culture medium. The yellow arrows indicate the distance from the edge of the silicone tubing to injected cells inside the tubing.
